# Post-traumatic-stress in the context of childhood maltreatment: pathways from attachment through mentalizing during the transition to parenthood

**DOI:** 10.3389/fpsyg.2023.919736

**Published:** 2023-06-09

**Authors:** Karin Ensink, Michaël Bégin, Gabriel Martin-Gagnon, Marko Biberdzic, Nicolas Berthelot, Lina Normandin, Peter Fonagy, Odette Bernazzani, Jessica L. Borelli

**Affiliations:** ^1^École de Psychologie, Université Laval, Québec, QC, Canada; ^2^Département de Psychologie, Université de Sherbrooke, Québec, QC, Canada; ^3^Department of Psychology, University of Wollongong, Wollongong, NSW, Australia; ^4^Department of Nursing, Université du Québec à Trois-Rivières, Québec, QC, Canada; ^5^Division of Psychology and Language Sciences, University College London, London, United Kingdom; ^6^Department of Psychiatry, Université de Montréal, Québec, QC, Canada; ^7^Department of Psychological Science, University of California, Irvine, Irvine, CA, United States

**Keywords:** attachment, mentalizing, RF, trauma, pregnancy, PTSS/PTSD

## Abstract

**Introduction:**

This study aimed to clarify the role of mentalizing in pathways from attachment to Post Traumatic Stress Symptoms (PTSS) in survivors of childhood maltreatment (CM). We focused on the transition to parenting, a critical period for reworking parenting representations to reduce intergenerational maltreatment cycles.

**Method:**

Study participants included 100 pregnant CM survivors. We assessed PTSS with the SCID and attachment and mentalizing with the Adult Attachment Interview (AAI), which was rated for Attachment and Reflective Functioning (RF).

**Results:**

Regarding Re-experiencing trauma symptoms, the results of the path analysis were consistent with mediation. CM survivors' mentalizing about their early relationships with their parents (RF-Other) directly impacted Re-experiencing trauma symptoms, and attachment had an effect on Re-experiencing trauma symptoms through mentalizing (RF-Other). Regarding Arousal/Reactivity symptoms, the results of the pathways analysis were consistent with partial mediation by mentalizing about early relationships with parents (RF-Other). In addition to the pathway from attachment via mentalizing (RF-Other) to Arousal/Reactivity, the pathway between attachment and Arousal/Reactivity also remained significant.

**Discussion:**

This study provides new evidence of a mentalizing and attachment model of PTSS in CM survivors. The findings indicate that increased mentalizing about early relationships with parents is an important process associated with lower PTSS. Finally, we discuss the implications of developing interventions for CM survivors to reduce PTSS. Scaffolding the development of mentalizing regarding attachment relationships in which CM occurred may help CM survivors reduce the intrusion of traumatic memories and decrease trauma-related arousal and reactivity symptoms. Interventions to help CM survivors mentalize regarding parents and attachment relationships in which trauma occurred may be particularly important during the transition to parenting when activation of representations of parenting can trigger PTSS.

## Introduction

Childhood maltreatment (CM) is a pressing public health concern impacting 25–55% of the population, depending on country and measurement (Moody et al., 2018). There is rapidly expanding evidence of the negative impacts of CM across the life course. Post-Traumatic Stress Disorder (PTSD) is the most frequent Axis I disorder associated with CM (Cloitre et al., 1997; Macpherson et al., 2021), with a 30–37% lifetime prevalence in adults with CM (Widom, 1999) compared to 5–10% in the community (Yehuda et al., 2015). For some CM survivors, Post-Traumatic Stress Symptoms (PTSS) can persist, unremitting, for years and decades. PTSS include intrusive recall of aspects of the event, avoidance of reminders, hyperarousal and hyper-vigilance, and dysphoria or anhedonia. In terms of a heuristic model, a dual-lens focusing on mentalizing and attachment in addition to trauma can elucidate risk and resilience processes for CM-associated PTSS (Lieberman and Amaya-Jackson, 2005; Pynoos et al., 2009; Ensink et al., 2021). As conceptualized by Fonagy et al. (2002), mentalizing references capacities involved in understanding attachment relationships and others, as well as oneself, in terms of mental states, affects, and intentions, thus enabling a mental perspective. In addition, mentalizing is a resilience factor in the context of trauma (Ensink et al., 2017; Duval et al., 2018). During pregnancy and the transition to parenthood, CM survivors are at heightened risk of PTSS (Muzik et al., 2013; Martinez-Torteya et al., 2014; Stacks et al., 2014; Choi et al., 2015; Christie et al., 2017). Further research is needed to inform theory and interventions with CM survivors to reduce PTSS and the intergenerational transmission of CM (Christie et al., 2017; Berthelot et al., 2018). However, there are few studies on the relationship between attachment, mentalizing, and CM-associated PTSS, especially during the transition to parenthood. To address current gaps in knowledge, we aimed to examine pathways involving attachment, mentalizing, and PTSS in pregnant women with CM.

### The clinical fall-out of maltreatment: PTSS

CM-associated impacts are evident across the lifecycle, including social difficulties, health-harming behaviors, mental and physical illness, increased allostatic load, reduced telomere length, and shorter lifespan (Rogosch et al., 2011; Bellis et al., 2019). In the context of CM, prolonged exposure to fear and anxiety triggers chronic and extreme activation of the stress response system (Heim et al., 2008; O'Donovan et al., 2011). Consistent with our focus on mentalizing regarding self and others, new PTSD diagnostic practices also include a focus on negative alterations in self-referential and other-referential cognitions in addition to symptoms (American Psychiatric Association, 2013; Friedman, 2013; Cox et al., 2014).

Pregnant women endure increased vulnerability to PTSS, and pregnant CM survivors are at even higher risk (Seng et al., 2010; Yildiz et al., 2017; Narayan et al., 2018, 2019). Increased PTSS vulnerability during pregnancy is likely because preparation for the caregiver role activates memories of the parenting they received (Slade et al., 2009; Ammaniti et al., 2013). In the context of CM, memories of abuse by caregivers were abusive is potentially re-traumatizing. However, pregnancy is also a critical period during which there are opportunities for reworking traumatic experiences and representations of parenting. Developing mentalizing regarding relationships with maltreating parents may promote better adjustment during this developmental transition by reducing PTSS so CM survivors can use the present to prepare for their future role as parents (Berthelot et al., 2018). However, there is a paucity of research to inform interventions to address CM and PTSS and help prepare future parents to reduce the risk of repeating patterns of abuse.

### Attachment and trauma

Parental responsiveness to infant distress establishes secure or insecure attachment (Bowlby, 1980) and calibrates the child's developing stress regulation system (Shai and Belsky, 2011, 2017; Mikulincer et al., 2015). Expectancies regarding the parent's availability to respond to distress become embedded in cognitive schemas and internal working models (Bowlby, 1973). For example, secure attachment involves representations of the self as deserving of care and others as available to help (Mikulincer et al., 2006; Fonagy et al., 2007).

Attachment and trauma impact similar physiological pathways involving fear and its regulation (Pynoos et al., 2009; Ensink et al., 2021), and attachment influences recovery after exposure to trauma. Consistent with this, secure attachment is associated with less severe PTSS in CM survivors, including adults (O'Connor and Elklit, 2008; Escolas et al., 2012; Ortigo et al., 2013), children (Ensink et al., 2021) and adolescents (Jardin et al., 2017). Conversely, insecure attachment is associated with more severe PTSS (Ogle et al., 2015; Woodhouse et al., 2015) and mediates (Muller et al., 2012) and moderates (Kanninen et al., 2003; Stovall-McClough and Cloitre, 2006; Sandberg, 2010; Ensink et al., 2021) the relations between CM and PTSS.

Maltreatment activates the attachment system so that children continue to seek out maltreating attachment figures, placing them in a situation of fear without resolution. This double bind leads to the approach/avoidance behaviors characteristic of disorganized attachment. In adults, disorganization is the mental state associated with CM (White et al., 2020), hypothesized to contribute to Unresolved Trauma and increased PTSS. For example, in CM-exposed psychiatric patients, Unresolved Trauma is associated with a seven-fold increase in PTSD (Stovall-McClough and Cloitre, 2006).

### Mentalizing: a potential mechanism

Mentalizing, operationalized as reflective functioning (RF) for research purposes, facilitates interpersonal functioning by making the reactions of others understandable and predictable (Fonagy et al., 2002) and aids stress regulation at a physiological level (Borelli et al., 2018). Difficulties in mentalizing are a transdiagnostic risk factor for psychopathology (Katznelson, 2014). Mentalizing develops optimally in relationships where children feel safe, and parents treat them as psychological agents whose behavior is motivated by mental states (Fonagy and Target, 2006; Ensink et al., 2017). Maltreatment stunts the development of mentalizing (Ensink et al., 2015, 2017). Furthermore, maltreatment likely installs an aversion to considering the minds of others, given the frankly destructive intentions toward them that the child must infer from the abusive act. Consisting with this theorizing, sexual abuse is associated with lower mentalizing regarding others (RF-Other; Ensink et al., 2015). Mentalizing regarding relationships with attachment figures (RF-Other) mediated the relationship between childhood sexual abuse and child depressive symptoms, as well as externalizing difficulties (Ensink et al., 2016a). In addition, mentalizing characterized by uncertainty about the reactions of others is associated with maltreatment and mediated the relationship between CM and personality disorder symptoms in adolescents (Duval et al., 2018).

In the context of CM, mentalizing and imagining their parents' psychological experiences and personal histories may help CM survivors gain a perspective that reduces the intrusion of past traumatic memories into the present. In line with this, Fonagy et al. (1991) showed in a seminal study that CM survivors who were able to mentalize regarding their attachment figures could establish secure attachment relationships with their infants. We replicated these findings in CM survivors and found that higher mentalizing regarding childhood maltreatment (RF-Trauma) was associated with a lower likelihood of infants having a disorganized attachment style (Berthelot et al., 2015). Furthermore, the relationship between CM and the quality of relationships with romantic partners and parenting behaviors was mediated by mentalizing about themselves (RF-Self: Borelli et al., 2020). In sum, different dimensions of mentalizing are associated with critical outcomes in the context of trauma.

### Current investigation

The present study aims to advance research literature by analyzing the relationships between attachment, mentalizing, and PTSS in CM survivors during the transition to parenthood. Within a cross-sectional design, we examined whether mentalizing regarding their early relationships with their parents empirically linked CM-exposed expectant mothers' attachment representations to their PTSS. Consistent with Fonagy's mentalizing model of attachment and mentalizing, we hypothesized that attachment would be associated with mentalizing regarding relationships with attachment figures (RF-Other). In turn, mentalizing would have a direct association with PTSS. This pathway, where attachment is linked to PTSS symptoms via mentalizing, would be consistent with mediation by mentalizing of the association between attachment and PTSS.

## Method

### Participants and procedure

This study used a subset of data from a longitudinal study regarding the intergenerational transmission of CM-related risk. We recruited pregnant women at the obstetrics clinic of a large metropolitan hospital in Canada and obtained informed consent for study participation. The hospital's ethics committee approved the study. Prospective participants (*n* = 809) were first screened at the hospital using the Parental Bonding Instrument (PBI; Parker et al., 1979) to identify women who had experienced inadequate parenting and maltreatment in childhood. Eligible mothers were over 18, were free from psychotic disorder or acute drug addiction, and lived within a 100 km range of the city. Of the 131 eligible participants, 101 women consented to study participation. We used the Childhood Experience of Care and Abuse (CECA) interview (Bifulco et al., 1994) to confirm CM. Participants completed the CECA, the SCID, and the AAI at their homes or the hospital. Postgraduate clinical psychology students and clinical psychologists conducted interviews. We recorded interviews for coding and reliability purposes and transcribed AAI interviews for subsequent coding.

The participants ranged in age from 18 to 41 years (*M* = 28.46, SD = 5.58). All women were pregnant at the time of the study; more than half (60%) had other children (*M* = 0.81, SD = 0.90). The sample was predominantly French-Canadian (73%), with the remainder being African–American (10%), Hispanic (6%), North African (4%), other Caucasian (4%), Asian (2%), and Native Canadian (1%). Approximately half (52%) of the sample were cohabiting, 34% were married, and 14% were single. Regarding education, 55% had post-secondary education, and 41% had been to university. The majority were employed (67%). Still, approximately half of the sample had an annual family income below $30,000, considered below the poverty index of roughly $34,000 for a Canadian family of one child.

Regarding CM, 58% reported physical abuse, 40% sexual abuse, 78% neglect, and 79% antipathy. A biological parent or primary caregiver perpetrated 79% of physical abuse, 38% of sexual abuse, 100% of neglect, and 100% of antipathy. Regarding the severity of CM, 59% of physically abused women experienced moderate to severe CM, 72% of sexually abused women experienced moderate to severe CM, 66% of neglected women experienced moderate to severe CM, and 67% of women who reported antipathy experienced moderate to severe CM.

### Measures

#### Screening measures

##### Parental care

The Parental Bonding Instrument (PBI; Parker et al., 1979) is a 25-item self-report questionnaire developed to assess adults' perception of parental care and the protection/control they received during the first 16 years of childhood. Respondents are questioned about their experiences with each parent separately to obtain care and protection scores for each parent. The instrument's psychometric properties have been extensively evaluated and shown to have good retest reliability, internal consistency, and validity (Parker, 1989, 1990), with demonstrated stability over twenty years (Murphy et al., 2010). The present study used the PBI as a screening instrument to identify pregnant women who had experienced low parental care from both parents in childhood. Standard PBI cut-off scores were care scores of 27 for maternal and 24 for paternal figures.

##### Child abuse

The Childhood Experiences of Care and Abuse interview (CECA; Bifulco, Brown and Harris,1994) is a semi-structured interview to measure adverse childhood experiences retrospectively. The CECA assesses different domains of childhood maltreatment experiences, including physical and sexual abuse, neglect, and antipathy. Ratings are made for each maltreatment type on a 1–4 scale (little/none, some, moderate, and marked) using a manual that provides explicit examples of the type of parental behavior considered to represent different levels of severity. The investigator-based format of the CECA has the advantage of not depending on participants to categorize their childhood experiences as abusive. In addition, the CECA has good psychometric properties, including inter-rater reliability and validity (Bifulco et al., 2005). In the current study, all CECA interviews were audiotaped and subsequently coded by trained raters. Doctoral students in clinical psychology administered and videotaped the CECA interviews. Two rates evaluated CECAs to confirm CM. All the mothers who screened positive on the PBI had confirmed cases of child maltreatment using the CECA.

#### Key study measures

Attachment. The Adult Attachment Interview (AAI; George et al., 1985) is a semi-structured interview designed to assess adults' state of mind regarding attachment relationships with their parents during childhood. Based on their general strategy evident in discussing attachment relationships, participants are categorized as secure-autonomous (F), insecure-dismissing (Ds), insecure-preoccupied (E), or cannot classify (CC), using Main et al. (2002) coding manual. Secure-autonomous individuals provide relatively clear, coherent, concise, and consistent responses. Individuals with adverse childhood experiences can be classified as secure-autonomous if they provide coherent accounts of adverse experiences showing that such experiences are processed. Insecure-dismissing participants, by contrast, give highly positive and idealized descriptions of their parents, contradicted later in interviews, and insist that they cannot remember experiences with their attachment figures. For insecure-preoccupied individuals, questions provoke excessive activation of attachment-related memories and confused, angry, or passive preoccupation with attachment figures. Participants who have experienced CM are assessed to determine whether they are unresolved/disorganized regarding CM (U/d). Lack of resolution is coded using a scale (1–9), with scores of 5 and higher considered to reflect lack of resolution (Main et al., 2002). Lack of resolution manifests in lapses in the monitoring of reasoning or discourse when individuals discuss traumatic experiences. We combined the Unresolved/disorganized and Cannot Classify classifications because of potential commonalities in etiology and sequelae (Bakermans-Kranenburg and van IJzendoorn, 2009). The AAI has good psychometric properties evidenced in high test–retest reliability, validity, and stability over time (Bakermans-Kranenburg and Van IJzendoorn, 1993; Benoit and Parker, 1994; Sagi et al., 1994).

In the present study, a rater trained to be reliable to the coding standards of the Berkeley laboratory of Mary Main and Erik Hesse coded AAI transcripts. The rater was naive to information regarding CM exposure. We used a three-way classification; Secure, Insecure (Including Dismissing and Preoccupied), and Unresolved/disorganized.

Reflective functioning. AAI transcripts were coded for RF using the RF manual (Fonagy et al., 1998). The RF coding system has good psychometric properties, including high inter-rater reliability and good discriminant, divergent, and predictive validity across samples (Fonagy et al., 1998; Taubner et al., 2013). RF is scored on a scale of −1 to 9, with higher scores indicating higher mentalizing. In addition, questions that explicitly demand an appreciation of mental states (e.g., “Why did your parents behave as they did during your childhood?”) are scored for RF. Two experienced raters trained by the developers of the RF coding system coded RF. Inter-rater reliability computed on the ten most complex cases (10% of the total sample) showed intraclass correlations of 0.79 for the RF ratings indicating good reliability on challenging transcripts. Mentalizing evident in the AAI can be scored in terms of participants' RF regarding early relationships with their parents (RF-Other), RF regarding self (RF-Self), and RF regarding trauma (RF-Trauma; Berthelot et al., 2015: Ensink et al., 2016b). We were specifically interested in RF-Other, as we hypothesized that RF regarding early relationships with parents would have the most significant implications for PTSS related to maltreatment by parents.

Post-traumatic stress symptoms. The Structured Clinical Interviews for DSM-IV Disorders I (SCID-I; First et al., 1997) was used to assess PTSS. Exposure to a traumatic event, re-experiencing the trauma, avoidance of the trauma-related content or situations, and hyperarousal/reactivity are assessed. Symptoms are rated on a three-point scale (not present, unsure, and present). The SCID has established reliability and validity (Zanarini and Frankenburg, 2001; Weertman et al., 2003). A clinical psychologist trained in SCID administration and rating conducted the interviews.

### Data analysis

First, we calculated descriptive statistics and correlations using IBM SPSS v.26. We intended to use the dimensions of RF significantly correlated with PTSS at the bivariate level in the path analysis. Next, we conducted a path analysis using Mplus 8.6 (Muthén and Muthén, 2017) in which we examined whether participants' attachment predicted their RF-O, which in turn predicted their PTSS. Specifically, we entered attachment using a three-level variable (secure, insecure, unresolved). For mentalizing, we selected participants' RF-Other, as the variable hypothesized to be the most relevant and the only RF dimension significantly correlated with PTSS. Next, we used PTSS Re-experiencing and PTSS Arousal scores assessed with the SCID for the outcome variable. Finally, using the maximum likelihood estimation method (ML), our model tested the indirect effects, which started from the predictor (attachment) to the PTSS outcomes (Re-experiencing and Hyperarousal) through mothers' RF-Other as a potential indirect effect. The indirect effects were bootstrapped 1000 times with 95% confidence intervals (CIs). To evaluate the fit of the model, we used the chi-square test, the Comparative Fit Index (CFI), the Tucker-Lewis Index (TLI), the root mean square error of approximation (RMSEA), and the standardized root mean square (SRMR).

Missing data were handled using the Full Information Maximum likelihood method (FIML). FIML enables using complete and incomplete observations and automatically adjusts the model estimation (Kline, 1998). FIML is superior to other missing data techniques, such as multiple imputations (Larsen, 2011).

## Results

### Descriptive statistics

Regarding attachment, participants were classified as Secure (*n* = 37, 36.7%), Insecure (*n* = 27, 26.7%), and Unresolved (*n* = 37, 36.7%). Correlations between the key study variables are presented in Table 1. Only RF (Other) significantly correlated with PTSS. Means and standard deviations for RF and PTSS by attachment type are presented as supplementary material.

**Table 1:** Correlation between attachment, mentalizing (measured as RF), and post-traumatic stress symptoms.

	**RF-self**	**RF-other**	**RF-trauma**	**PTSS Re-experiencing**	**PTS avoidance**	**PTS hyperarousal**
Attachment (AAI)	−0.21^*^	−0.29^**^	0.738^**^	0.22^*^	0.23^*^	0.26^*^
RF-self	1	0.87^**^	0.10	−0.05	0.09	0.00
RF-other	–	1	−0.04	−0.31^**^	−0.06	−0.24^*^
RF-trauma	–	–	1	0.17	0.20	0.232
PTS Re-exp	–	–	–	1	0.80^**^	0.92^**^
PTS avoidance	–	–	–	–	1	0.85^**^
PTSHyperarousal	–	–	–	–	–	1

Note: Adult Attachment was assessed as Secure, Insecure, Unresolved. Mentalizing was assessed as RF-Self, RF-Other and RF-Ttrawna, PTSS was assessed with the SCID, Re-experiencing, Avoidance of trauma related content or situations, Hyperarousal

* p < 0.05.

** p < .O1.

### Path analysis

We conducted a path analysis to test the mediational effect of RF-Other. In other words, the analysis tested whether attachment predicted RF-Other, which predicted PTSS.

**Figure 1:**
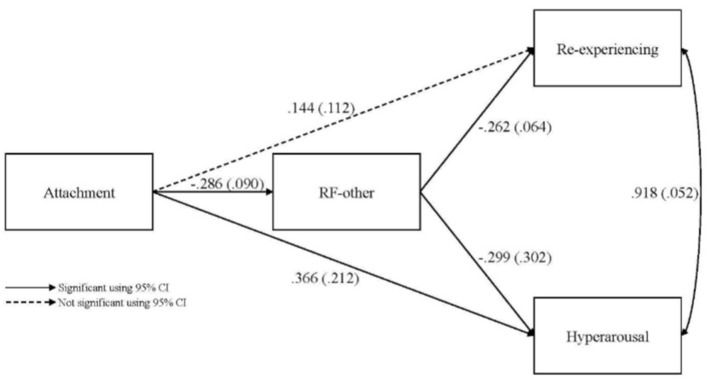
Path analysis from attachment to post-traumatic stress symptoms with mentalizing (RF-other) as a mediator.

The results of the path analysis showed that RF-other indirectly linked attachment (Secure, Insecure, Unresolved) and PTSS Re-experiencing and Hyperarousal/Reactivity symptoms. Fit indices indicated that the hypothesized model was a good representation of the observed data, χ^2^ (1) = 0.002, *p* = 0.999, χ^2^/*df* = 0.002, CFI = 1.00, TLI = 1.04, RMSEA = 0.00 and SRMR = 0.00. Because of the 3-level attachment variable, regression coefficients reflect the mean slope from one level to another (mean of the slopes between 1 and 2 and between 2 and 3). The full results of the path analysis are reported in Figure 1. Regarding attachment and RF-Other, results showed that more insecure and disorganized attachment were associated with lower levels of RF-Other (*β* = −0.286, *p* = 0.001). The model explained 8.2% of the variance of RF-other. Only RF-Other, not attachment, was significantly associated with Re-experiencing symptoms (*β* = −0.262, *p* < 0.001). The indirect effect of attachment on Re-experiencing symptoms through RF-other was also significant (*b* **=** ***0.0***75, 95% CI [.022, 0.162]). The indirect effect (*b* = 0.075) accounted for 34.2% of the total effect of attachment on Re-experiencing symptoms. Once RF-other was included in the model, the direct effect of attachment was no longer significant (*β* = 0.144, *p* = 0.100). This is consistent with full mediation by RF of the relationship between attachment and Re-experiencing symptoms. The model explained 11.2% of the variance in Re-experiencing symptoms. The Re-experiencing symptoms curve was flat from secure to insecure attachment, with a steeper increase from insecure attachment to unresolved trauma. However, the direct effect of attachment was not significant.

Concerning Hyperarousal/Reactivity symptoms, both attachment (*β* = 0.366, *p* = 0.015) and RF-Other (*β* = −0.299, *p* = 0.033) were associated with PTSS. More insecure and Unresolved/disorganized attachment (where attachment was rated as Secure, Insecure, and Unresolved/disorganized) and lower RF-Other were associated with higher Hyperarousal/Reactivity symptoms. Furthermore, the indirect effect of attachment on Hyperarousal/Reactivity through RF was significant (*b* **=** ***0.0***86, 95% CI [.033,0.140]). The indirect effect (*b* = 0.086) accounted for 18.7% of the total effect. The direct effect of attachment (*β* = 0.366) on hyperarousal remained significant. The model explained 9.4% of the Hyperarousal/Reactivity symptoms variance. The Hyperarousal/Reactivity symptoms curve was flat from secure to insecure attachment with a steeper increase from insecure attachment to unresolved attachment, suggesting that the latter attachment type contributes the most to the significance of the observed coefficients.

## Discussion

The present study aimed to investigate pathways to PTSS involving mentalizing and attachment in pregnant CM survivors. As hypothesized, higher PTSS was associated with lower mentalizing regarding early attachment relationships (RF-Other). Furthermore, the findings of the path analysis were consistent with mediation via RF-Other of the effect of attachment on Re-experiencing symptoms and partial mediation of the effect of attachment on Hyperarousal/reactivity symptoms. The study provides new evidence supporting a mentalizing and attachment model of CM-associated PTSS. The findings have important clinical implications suggesting that increasing mentalizing about attachment relationships, including those in which maltreatment occurred, may decrease PTSS.

The study findings lend further credence to the now robust observation that mentalizing is frequently deficient in the context of PTSS and CM (Nietlisbach et al., 2010; Mazza et al., 2012; Nazarov et al., 2015; Palgi et al., 2016). In addition, it adds to the growing body of research showing that mentalizing is a resilience factor in the context of CM that may reduce the risk of subsequent psychological difficulties (Berthelot et al., 2015; Ensink et al., 2016a, 2017; Duval et al., 2018). The findings regarding mediation by RF-Other extend previous research by Huang et al. (2020), who, using self-report measures, found that mentalizing difficulties characterized by uncertainty and attachment insecurity mediated the effect of childhood trauma on PTSS. By using observer-based measures of PTSS, mentalizing, and attachment, the current study revealed the salience of mentalizing regarding attachment relationships (RF-Other) for CM-associated PTSS.

Regarding attachment and Hyperarousal/Reactivity symptoms, mediation by mentalizing was partial, and the effect of attachment remained significant. To understand the effect of attachment on PTSS further, we examined the attachment and PTSS curve. The curve was flat from secure to insecure attachment, with a steeper increase from insecure attachment to unresolved trauma, suggesting that unresolved trauma contributes the most to explaining variance in PTSS symptoms. These findings are consistent with research by Stovall-McClough and Cloitre (2006), showing that unresolved trauma was associated with a seven-fold increase in PTSS in hospitalized CM survivors.

Our findings have important implications for intervention and suggest that improving mentalizing about early attachment relationships may reduce Re-experiencing and Hyperarousal/reactivity in pregnant CM survivors. Mentalizing about early attachment relationships with parents may help CM survivors reduce the intrusion of traumatic memories and hyperarousal. During pregnancy, CM survivors may benefit from interventions that help them look back and rework traumatic relationships without becoming re-traumatized. Such intervention may help reduce the intrusion of memories of past trauma into the present so that CM survivors can use the present to prepare for the future and becoming parents themselves (Berthelot et al., 2018).

While we did not examine Complex PTSD, our findings regarding long term CM-associated PTSS are consistent with a Complex PTSD framework. The findings expand our understanding of PTSS, mentalizing, and attachment processes in CM survivors and show the importance of mentalizing about early attachment relationships (RF-Other), especially those in which abuse occurred, in pathways to PTSS. This extends previous research showing that, in CM survivors, RF-Other mediated the relationship between sexual abuse and psychological difficulties (Ensink et al., 2016a). We did not find associations between PTSS and mentalizing regarding trauma (RF-Trauma) nor mentalizing regarding self (RF-Self). However, previous research shows that RF-Trauma has implications for other outcomes in CM survivors, such as investment in their pregnancy (Ensink et al., 2014) and infant attachment organization (Berthelot et al., 2015).

Furthermore, RF-Self was previously shown to mediate the relationship between insensitive care in childhood, romantic relationships with partners, and maternal behavior in CM-exposed parents (Borelli et al., 2020). While our findings highlight the importance of mentalizing about attachment relationships (RF-Other) for reducing PTSS in CM survivors, it complements previous research showing that mentalizing about self (RF-Self) and trauma (RF-Trauma) also affect psychological adjustment, romantic and attachment relationships, intergenerational transmission of risk, and resilience in CM survivors. The complementary findings are consistent with mentalizing being a multicomponent construct, with some mentalizing difficulties contributing to psychological difficulties and other mentalizing difficulties impacting relationships with partners and infant attachment.

The finding that lower mentalizing was associated with more PTSS is also in line with existing evidence that among CM survivors mentalizing difficulties are transdiagnostic risk factors for a range of psychological difficulties, including personality disorder features (Chiesa and Fonagy, 2014; Duval et al., 2018), depressive symptoms, dissociation, externalizing and sexualization (Ensink et al., 2016a, 2017). To date, empirically supported treatments such as mentalization-based treatment (MBT; Bateman and Fonagy, 2008) are effective for patients with personality disorders (Storebo et al., 2020; Stoffers-Winterling et al., 2022). MBT is being adapted for treating CM-associated PTSD to address the difficulties CM survivors experience in emotion regulation and interpersonal relationships. Furthermore, Berthelot et al. (2018) have developed prenatal interventions to facilitate mentalizing about parenting and past trauma specifically for CM survivors.

The study has definite strengths, such as observer-rated measures of attachment and RF and PTSS. However, the study has limitations that need consideration before generalizing based on the findings. The sample size is adequate given the low number of latent variables and indicators, the absence of missing data, and Monte Carlo analyses showed that the sample size provided sufficient power. However, further studies using larger samples are needed. All study participants were pregnant women and replication with a gender-diversified sample would enable testing whether the findings apply to men. We were particularly interested in understanding the relations between PTSS, mentalizing, and attachment during pregnancy to inform interventions during this critical preparation period of transition and preparation to becoming parents. Including measures of pregnancy-specific processes, such as attachment to the fetus, could have enhanced the study further. Also, the sample was diverse regarding socio-economic status, but replication in higher-risk samples is needed. Many study participants experienced moderate to severe physical and sexual abuse. However, replication in a larger sample is required before we can generalize the findings to different CM types and severity.

Furthermore, our focus on PTSS advances our understanding of CM's long-term impacts and is consistent with a complex PTSD framework, but including a measure of complex PTSD can enhance the contribution of future studies. Finally, we cannot optimally establish mediation, given that we simultaneously assessed our independent variable, mediator, and dependent variables. To optimally test the proposed theoretical models offered within this study, one would need to assess attachment at the first time point, RF at the first and subsequent time points, and the dependent variable at the second and third time point. The statistical model would need to incorporate the final time points of all measures while controlling for prior assessments of these measures.

## Conclusion

This study provides new evidence supporting a mentalizing and attachment model of PTSS in CM survivors. For CM survivors, mentalizing about early attachment relationships (RF-Other) may be particularly important for reducing Re-experiencing and Hyperarousal/reactivity. The findings have important clinical implications. Interventions to scaffold mentalizing about their parents and attachment relationships in which abuse occurred (RF-Other) could help CM gain mental perspective and reduce the intrusion of traumatic memories and hyperarousal.

## Data availability statement

The original contributions presented in the study are included in the article/supplementary material, further inquiries can be directed to the corresponding author.

## Ethics statement

The studies involving human participants were reviewed and approved by Ethics Committee of Montreal University. The patients/participants provided their written informed consent to participate in this study.

## Author contributions

All authors listed have made a substantial, direct, and intellectual contribution to the work and approved it for publication.
